# Role of Laparoscopy in the Management of Neonatal Ovarian Cysts

**Published:** 2014-04-01

**Authors:** Vijay C Pujar, Shirin S Joshi, Yeshita V Pujar, Hema A Dhumale

**Affiliations:** 1 Department of Pediatric Surgery, KLE University, Belgaum, Karnataka; 2 Department of Obstetrics and Gynecology, KLE University, Belgaum, Karnataka

**Keywords:** Neonate, Ovarian cyst, Laparoscopy

## Abstract

Aim: Ovarian cysts in the newborn period are simple cysts which resolve on their own. Complications like torsion leading to loss of ovarian units are well documented. Surgical treatment should always be performed in a way to protect the ovaries and to ensure future fertility. The aim of this study was to study the safety and efficacy of laparoscopic management of neonatal ovarian cysts.

Materials and Methods: Neonates with ovarian cysts over last 6 years were retrospectively studied. Thirty seven ovarian cysts were detected antenatally and 32 of them persisted postnatally. All babies were asymptomatic. Nine babies with cyst size more than 5cms underwent laparoscopic deroofing, fenestration or cystectomy; the rest 23 babies were managed conservatively. Sonographic monitoring was done at monthly interval for change in contents, echogenicity of walls and features of torsion. Follow up was done with USG at 3 and 6 months and MRI after 1 year.

Results: No procedure related complications were seen in the laparoscopy group and no loss of ovarian units were seen in 1 year follow-up. In the observation group, cysts resolved in 3- 12 months period. Three babies developed complications and 4(17%) ovarian units were lost.

Conclusion: Ovarian cysts are the most frequent among intra-abdominal cysts in newborns. Neonatal ovarian cysts are known to resolve spontaneously. Laparoscopic management of these cysts is safe and efficacious even in neonatal age and should be the treatment of choice when indicated.

## INTRODUCTION

The incidence of neonatal ovarian cysts is reported as high as 34%.[1] Neonatal cysts are thought to be the result of stimulation of the fetal ovary by placental chorionic gonadotropin causing follicular dysgenesis.[2-4] Because of the decrease in hormonal stimulation that occurs after birth, ovarian cysts, especially small ones, generally regress spontaneously.[5,6]

Laparoscopic management provides correct diagnosis in newborn ovarian pathologies and allows therapeutic interventions including aspiration, fenestration, cyst excision, or oophorectomy. The magnitude of the cyst size is not a contraindication, since laparoscopic cyst puncture and aspiration allows sufficient reduction in size.[6,7] Laparoscopy may be safely performed even in neonates and offers advantages associated with minimally invasive surgery. [8] [8]

## MATERIALS AND METHODS

The study was conducted at KLE Dr Prabhakar. Kore Hospital and MRC Belgaum over a period of 6 years from 2006 to 2011. The inclusion criteria were neonates with ovarian cysts diagnosed antenatally which persisted postnatally. Infants referred beyond 1 month of age or neonates with complex cysts were excluded from the study.

They were divided in two groups depending on the size of cysts assessed postnatally. Cysts more than 5 cms underwent laparoscopy in the newborn period and those with less than 5cms were observed for a period of 1 year. Neonates in laparoscopy group underwent laparoscopy by inserting 3mm camera port at umbilicus by open Hassan’s technique. One or two working ports were placed in the flanks depending on nature of cyst and the treatment planned. Percutaneous needle aspiration, deroofing, fenestrations or cystectomy were done.

Cysts less than 5cms size (observation group) were monitored sonographically at 3 monthly interval for a period of 1 year. Change in size, contents, echogenicity of walls or features of torsion were noted.

## RESULTS

Thirty seven neonates with ovarian cysts were detected antenatally, out of which 32 of them persisted postnatally. All neonates were asymptomatic. Cysts size more than 5cms were seen in 9 babies who underwent immediate intervention with laparoscopy. Remaining 23 babies with cyst size less than 5cms were grouped as observation group. Both groups were followed up to a period of 1 year. They underwent sonography at 3 monthly interval and MRI at 1 year of age.

**Figure:**
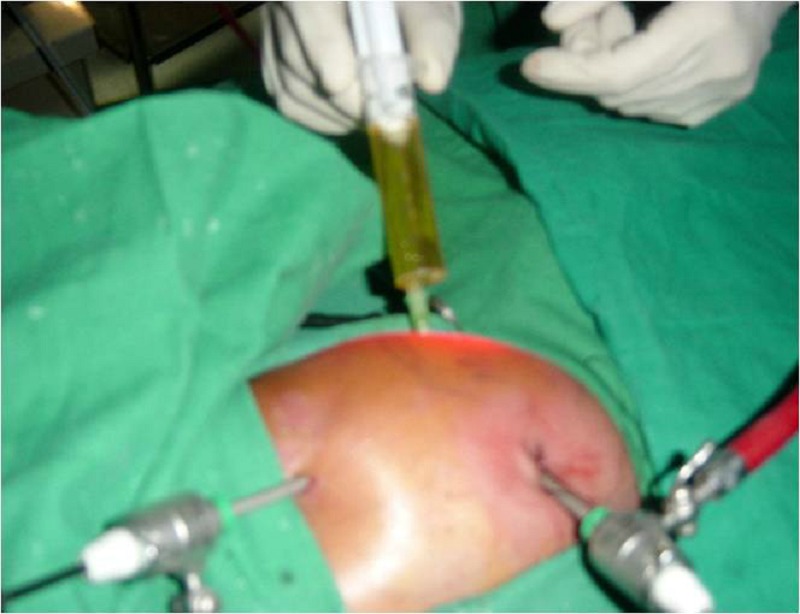
Figure 1: Ovarian cyst aspiration under laparoscopic guidance

There were no procedure related complications seen and no ovarian units were lost in the laparoscopy group. The mean operating time was 34 minutes. The detail of the procedures is as shown in table 1.

**Figure:**
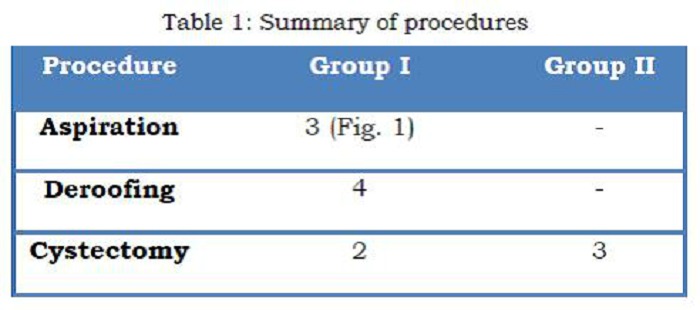
Table 1: Summaries of Procedures

In the observation group, the cysts resolved in 3-12 months. Three babies noticed to have developed complications in the follow up period and the laparoscopy revealed torsion that led to loss of ovaries (17%). Two had auto amputation and one had intra-cystic bleed.

## DISCUSSION

Fetal ovarian cysts were first described as a prenatal finding by Valenti et al in 1975 and were believed to be a rare finding. Now the incidence of neonatal ovarian cysts is reported as 34%.[9] On the basis of their sonographic features, these cysts are divided into two types: simple and complex or complicated. Simple ovarian cysts tend to resolve spontaneously, which is known to occur in 25% to 50% of cases in the first year of life.

These cysts arise from ovarian follicles and are thought to be stimulated by the hormonal effects of fetal follicle-stimulating hormone, maternal estrogens, and placental human chorionic gonadotropin. Approximately 50% of the simple cysts will resolve spontaneously after birth. This probably occurs secondary to acute changes in the hormonal environment after birth, with a marked decrease in maternal estrogen and placental human chorionic gonadotropin and the inhibition of follicle-stimulating hormone secretion in the fetus by the hypothalamic-pituitary-ovarian axis. Fetal ovarian cysts are unilateral in 95% of cases and can have simple or complex characteristics. Simple cysts are completely anechoic, whereas complex cysts may contain internal echoes, fluid levels, septations, or echogenic foci.[10]

The differential diagnosis includes choledochal, mesenteric, urachal and enteric duplication cysts, hydrometrocolpos and lymphangioma.[11]

Most simple cysts measuring less than 4 cm do not develop complications and resolve within the first 6 months as hormonal stimulation decreases.[12]

Torsion is the most common reported complication bearing an incidence of 50% to 78% of all neonatal ovarian cysts. The risk of torsion is said to correlate with the size of the cyst. The risk is higher for cysts greater than 4 to 5 cm in size.[1,5,7,13] Antenatal torsion occurs in 20-32% of cysts.[14] Other rare complications include gastrointestinal obstruction or perforation, urinary tract obstruction, incarceration of an inguinal hernia, ovarian auto-amputation, and sudden infant death. 

Treatment options include expectant management, antenatal or neonatal cyst aspiration, laparoscopic cystectomy, and laparotomy. 

Timely surgical intervention is necessary for large, uncomplicated cysts and should take place within the first few days after delivery to prevent loss of ovarian tissue. Ultrasound-guided aspiration for large simple cysts appears as simple solution as it avoids general anesthesia, but is not definitive as repeated aspirations may be necessary, which increases the possibility of bleeding and infection in the cyst. In 18 neonates included in our study (56%), the diagnosis was not clear.

Laparoscopic removal of a neonatal ovarian cyst was first described in detail in 1995.[15] We conducted laparoscopy in the first week of life, hence loss of ovarian units were not seen in laparoscopy group. Whereas removal of the ovary was required in 7 (31%) cases of observation group as complications were noted in follow up period. Cystectomy or removal of amputated cysts was done. Advantages of laparoscopy are excellent visualization of the contralateral ovary, rapid postoperative recovery, and excellent cosmesis.

## Conclusion

Laparoscopic management provides correct diagnosis in newborn ovarian pathologies and allows therapeutic interventions including aspiration, fenestration, cyst excision, or oophorectomy.Large ovarian cysts >5 cms are prone for more complication and early laparoscopic procedure helps in salvaging these ovarian units. 

## Footnotes

**Source of Support:** Nil

**Conflict of Interest:** None

